# Spatial and temporal distribution of incidence of acquired equine polyneuropathy in Norway and Sweden, 1995–2012

**DOI:** 10.1186/s12917-014-0265-9

**Published:** 2014-11-15

**Authors:** Cecilia Wolff, Agneta Egenvall, Siv Hanche-Olsen, Gittan Gröndahl

**Affiliations:** Department of Clinical Sciences, Swedish University of Agricultural Sciences, PO Box 7054, SE-75007 Uppsala, Sweden; Norwegian School of Veterinary Science, Equine Section, PO Box 8146 Dep, N-0033 Oslo, Norway; Department of Animal Health and Antimicrobial Strategies, National Veterinary Institute, 751 89 Uppsala, Sweden

**Keywords:** Acquired equine polyneuropathy, Knuckling, Horse, Space-time scan statistics, Space-time K-function, Jacquez k nearest neighbour test

## Abstract

**Background:**

Acquired equine polyneuropathy (AEP) is an emerging disease in horses in Sweden, Norway and Finland since 1995. Affected horses show bilateral pelvic limb knuckling and weakness, sometimes progressing to recumbency and euthanasia. The aetiology is unknown but is thought to be non-infectious and non-genetic, though possibly toxic or toxico-infectious. The objectives of this study were to describe the spatial, temporal and spatio-temporal features of AEP in Norway and Sweden for the period of 1995 to 2012. Data from all documented case farms (n = 136) were used. Space-time interaction clustering of case farms was investigated with a retrospective space-time scan statistic with a space-time permutation model, the space-time K-function and the Jacquez k nearest neighbour (kNN) test.

**Results:**

There was a clear seasonality in disease occurrence, as 123 case farms presented their first case from January to May. However, there was large variation in the number of case farms between years. Case farms were more numerous in certain regions. Despite the larger horse population in Sweden, 120 of the case farms were in Norway. Space-time clustering was supported by the K-function and partly by the space-time scan, but not by the Jacquez k nearest neighbour (kNN) test.

**Conclusions:**

The results suggest an aetiology for AEP where the exposure is not consistent in time, but varies during and between years, assuming that the incubation period does not vary greatly. The results further suggest that the exposure varies between regions as well. Two out of three different analytical methods supported spatio-temporal clustering of case farms, which rendered inconclusive results. The negative result in the kNN test might be explained by lack of power, which is due to the small number of outbreaks in relation to the size of the study area and length of the study period, and further by the low to moderate power of methods to detect space-time clustering when the background population is unknown. Further research is needed to study how management, meteorological variables and other factors with local or regional differences may explain outbreaks of AEP.

## Background

Acquired equine polyneuropathy (AEP), sometimes referred to as “Scandinavian knuckling syndrome”, is an emerging disease in horses in Sweden, Norway and Finland since 1995 [1–4]. Nerve lesions consisting of a mixed axonal and demyelinating polyneuropathy with inflammatory features and Schwann cell hypertrophy with cytoplasmic inclusions have been described [2,3]. Except for case reports from Japan on polyneuropathy with knuckling in horses, but with different clinical and pathological features [5,6], similar cases have not been described in the literature, and the aetiology and the pathogenesis are still unclear.

Clinically, AEP typically presents with signs of bilateral pelvic limb knuckling. Extreme weakness leading to recumbency has been reported in 33 out of 75 cases in one study [3] and in ten out of 42 cases in another [4]. More than one horse in the same establishment is often affected. In one study of 13 affected farms, all cases on a farm appeared within 100 days of the index case [4]. The horses are bright, alert and responsive and there are no signs of general infection or central nervous involvement [4]. Case fatality is reported to be 29–53% [3,4], and is mostly due to severe clinical signs leading to recumbency and euthanasia. In two studies, less affected cases recovered from all signs and returned to previous work level within 19 months [3,4].

The aetiology of AEP is unclear but is thought to be non-infectious. The case material in epidemiologic studies does not indicate a genetic trait [3,4]. A toxic or toxico-infectious aetiology for AEP is possible, involving exposure of horses to a common factor, possibly from the forage: reported cases had usually been fed wrapped forage (haylage or silage) [1,3,4].

To study the distribution of disease in space and time is one of the basics of epidemiology. Cases of AEP have occurred mainly during late winter to early spring [3]. To our knowledge, only Nordic countries have reported AEP, with most cases having been found in Norway.

If disease cases occur in closer succession and in a smaller area than would be expected by chance alone, they are said to cluster in space and time. This appears in outbreaks of infectious diseases, e.g. acute respiratory disease in cattle [7], infectious bursal disease in broiler flocks [8], and sheep scab [9]. But also non-communicable diseases in humans, such as diabetes [10] and gliomas [11], have been shown to cluster in space and time.

If farms with AEP cases were found to cluster in space and time this might be suggestive of an underlying but transient risk factor that is shared by the clustering farms. Knowledge of such factor would help in generating hypotheses about the aetiology for AEP. Therefore, the objectives of this study were to describe the spatial, temporal and spatio-temporal features of AEP in Norway and Sweden for the period of 1995 to 2012.

## Methods

### Study population and data collection

A retrospective study of all recognised cases of AEP in Norway and Sweden during 1995–2012 was initiated. Inclusion criteria for AEP cases were signs of digital extensor dysfunction in the pelvic limbs with consistent repeated knuckling, and otherwise normal behaviour, appetite and alertness at clinical examination. Exclusion criteria were ataxia or neurological signs indicating brain involvement. Cases and case farms were identified for inclusion in the study when attending veterinarians or owners contacted the Equine Clinic at the Norwegian School of Veterinary Science, the Equine Clinic at the Swedish University of Agricultural Sciences or the Swedish National Veterinary Institute for information, referrals or post-mortem examinations. During the study period, repeated publicity on the outbreaks increased the public awareness of AEP in both countries.

The data collection was mostly passive with regard to identifying case farms. It was active with regard to collecting information about the cases. The first horse showing signs of AEP at each farm was defined as the index case, and the month of the first observation of AEP signs was denoted as the index month of each outbreak. The number of cases on each case farm was retrieved. Data were entered into a spreadsheet.

The case farms were geo-referenced with coordinates retrieved from phone directories of Norway and Sweden (www.gulesider.no; www.gulesider.nowww.eniro.se). Where the exact address was unknown, the church or another clearly defined feature in the nearest village or city was used as georeference point. Farms located in the same village but without exact address were provided with references, with slight differences between individual references to enable separation in the analyses. Map shape files of Norway and Sweden were retrieved from DIVA_GIS (www.diva-gis.org). All geographic data were transformed to the projection RT90. Unless otherwise specified, data management and analyses were done in the R statistical package version 3.0.1 [12].

### Temporal description

Epidemiological curves with the number of case farms per year and per calendar month were created to describe the temporal characteristics of the outbreaks.

### Spatial description

Spatial data, i.e. geographical (point) locations of case farms, were plotted on a map of Norway and Sweden.

### Space-time description methods

To investigate space-time clustering, the null hypothesis was spatio-temporal randomness, i.e. that case farms were distributed without clustering in space and time. The study population in an exploratory spatio-temporal study does not necessarily include all cases but is assumed to represent the population of cases present in the study area and study period. If cases are compared with controls or the entire background population in the same study area and study period, any apparent space-time clustering that is in fact caused by heterogeneity in the underlying population structure is dealt with. Where information on the underlying population structure or controls is unavailable, methods that evaluate only the location of the case data and adjust for purely temporal as well as purely spatial variation in the data can be used [13–16]. This was the case in the present study as information on the geographical (spatial) distribution of the horse population in Norway was not available, while for Sweden it was only available at the county level and only with data assembled with varying methods. Also, the temporal changes of the horse populations in Norway and Sweden were unclear.

The explicit localisation and size of clusters in space and time can be identified and statistically tested with specific (“local”) cluster detection methods. The overall characteristics of the data across the entire study area and study period are not described by such methods. Such characteristics can, however, be explored with non-specific (“global”) cluster detection methods. These test whether there is space-time interaction clustering, in general, of cases in the data, but do not give the localisation or size of any clusters [13]. In this study, one local and two global methods were used to explore the spatio-temporal features of AEP case farms. These methods do not evaluate purely spatial or purely temporal clustering of cases.

### Local detection of space-time interaction clusters

Specific cluster detection, i.e. detection of the location and size of any clusters of AEP, was carried out using a retrospective space-time scan statistic [17] with a space-time permutation model [14]. In brief, the study area was scanned with a moving search window that could be enlarged or reduced. A cylindrical scanning window was used, where the size of the base circle was the geographical search area and the height of the cylinder was the time frame. The shape of the cylinder therefore varied from wide and low, i.e. covering a large geographical area but short time period, to narrow and high, i.e. covering a small geographical area but long time period, and all combinations in between those two. The observed number of cases inside of the search window was compared with the number of cases outside the window. The maximum spatial window was allowed to include 50% of the study population while the temporal window was allowed to include up to 50% of the study period [18]. Analysis was first done at the case level where each case farm location was weighted by the number of affected horses per farm. Case farms where this information was not available were excluded in this analysis. Analysis was also done at case farm level, without regard to the number of cases per farm, i.e. each case farm was included as one case. For both case and case farm level analyses the search was for clusters of either a higher or a lower number of cases than expected. No geographical overlap of clusters was allowed.

Because of the long study period and the lack of data on how the distribution of horses in Norway and Sweden had changed, the scan statistic was, in addition, run with the study period divided into three shorter periods, namely 1995–2000, 2001–2006 and 2007–2012. This was done to avoid identification of false clusters caused by heterogeneous changes (in space and over time) of the underlying population. [13].

To test whether the risk of AEP was the same inside as outside of the scanning window, a likelihood ratio test was run with 999 Monte Carlo iterations for each location and size of the scanning window. In each permutation the time of the data points was rearranged among the geographical position of the points [18]. The SaTScan package [19] was used.

### Global tests of space-time interaction clustering

Both these tests were at case farm level, i.e. each case farm was one case. In the first method, the space-time K-function, described in detail by Diggle et al. [15], was used. In brief, the space-time clustering was estimated as the observed spatio-temporal point pattern relative to a pattern that had the same spatial and temporal properties (events per unit) as the original data, but no space-time clustering, i.e. the cases occurred independently in time and space. The space-time K-function (K(s, t)) is defined as the expected number of cases that occur within spatial distance *s* and temporal distance *t* from a randomly selected case. If there is no space-time clustering, K(s, t) is the product of the space K-function (K(s)) and the time K-function (K(t)).

Next, the difference function D(s, t), defined as the difference between K(s, t) and K(s)*K(t), was calculated. If D(s, t) >0, this indicates the presence of space-time clustering. The higher the D(s, t) the stronger the evidence. Because D(s, t) naturally increases with longer distances and time, a corrected variable, D_0_(s, t), is calculated as D(s, t)/ K(s)*K(t). D_0_(s, t) is interpreted as the proportional increase or excess risk attributable to space-time clustering. Values of D_0_(s, t) >1 indicate at least a doubling in risk, i.e. that the number of observed events was more than twice the number of expected events. In the present study, the K-function was estimated over a space-time grid of 100 km times 12 months. The values of D_0_(s, t) were plotted as a surface over this space-time grid to illustrate any elevated disease risk.

To test the null hypothesis of no space-time clustering, the index months of the case reports were randomly rearranged among the fixed set of case locations, using Monte Carlo simulation with 999 replications, generating a distribution of D(s, t) values. The sum of D(s, t) from the observed data was compared with the simulated distribution. A p-value was calculated as the proportion of simulated observations that exceeded the observed value.

The analyses were also repeated with subsets of the data, covering the study years 1995–2000, 2001–2006 and 2007–2012. In addition, K(s, t) was estimated for a study area limited to southern Norway, where most cases of AEP were reported. As with the scan statistic, this was done to reduce any possible bias of the estimate of K(s, t) by any heterogeneous changes in the background population. These analyses were carried out with the splancs package for R version 2.01-32 [20].

In the second global method, the Jacquez k nearest neighbour (kNN) test was carried out [16]. The kNN test also evaluates the presence of space-time clustering, but is based on nearest neighbours instead of the actual distances in space and time between the locations of case farms. The first test statistic is J_k_, which is the number of case pairs that are k (a positive integer) nearest neighbours in both space and time. J_k_ increases when there is space-time clustering. If there is no space-time clustering, J_k_ is small, and the probability of two cases being nearest neighbours in time is not dependent on how they are related spatially. Because J_k_ will increase also when k is increased, ΔJ_k_ is also calculated as the k-specific increase in space-time nearest neighbours when k is increased by 1. Thus, the cumulative effect of increasing k is removed. Unlike the K-function, the kNN test does not give the magnitude of any clustering, but has the advantage that temporal and spatial distances do not need to be defined prior to analysis.

Similarly to the K-function, the test statistics were evaluated by creating reference distributions where the times (index month) were randomised across the spatial locations of case farms with 999 iterations. The probabilities of the observed values that would occur by chance were calculated by comparing the observed J_k_ and ΔJ_k_ with their reference distributions. The test statistic was performed for k 1–10 and statistical significance was evaluated for each level of k and combined for all levels of k. Sime’s correction to the Bonferroni procedure was used to account for interaction over several levels of k for the combined p-value. The analysis was performed with ClusterSeer® [21].

The study did not need approval of an ethical committee.

## Results

In the study period 1995–2012, the study population included 136 case farms affected with AEP, in which detailed geographical location was available for 135 farms. Index month was known for 135 case farms, and information on the number of case horses (i.e. horses with signs of AEP) on each farm was available for 118. In total there were 334 cases on these 118 case farms. The median (first quartile, third quartile) number of cases per case farm was 2 (1, 3.75) and the range 1–25. No farm had more than one reported outbreak.

### Temporal description

**Figure 1:**
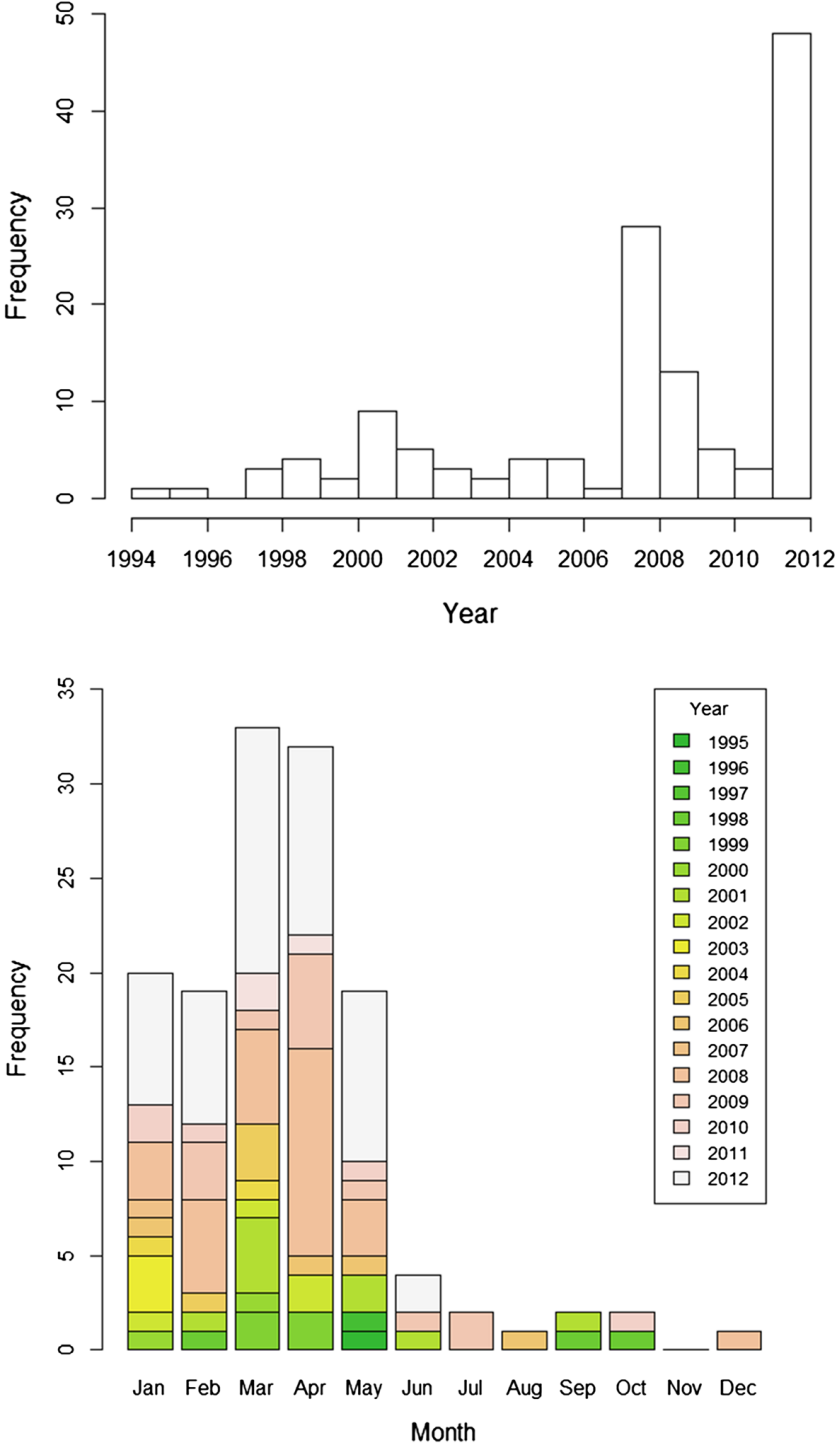
**(top) Yearly and (bottom) monthly frequencies of case farms with acquired equine polyneuropathy.** In a retrospective study of all farms (n = 136) with at least one reported case in Norway and Sweden in 1995–2012. The month in which the first case was noted at a farm was used as the index month of the outbreak for that farm (n = 135).

There were reported cases from NO every year of the study period (1995–2012) except in 1997. From SE, cases were reported in year 1998, 1999, 2001, 2005, 2006, 2008 and 2009 (Figure 1). The median (first quartile, third quartile) number of case farms per year was 4 (2, 5). Seasonality was obvious, with 123 out of 135 case farms (91%) having their index case appear in January–May (Figure 1). The remaining index cases were distributed across the remaining months, but there were no cases in November (Figure 1). The median (first quartile, third quartile) number of case farms per month during the study period was 1 (1, 3).

### Spatial description

**Figure 2:**
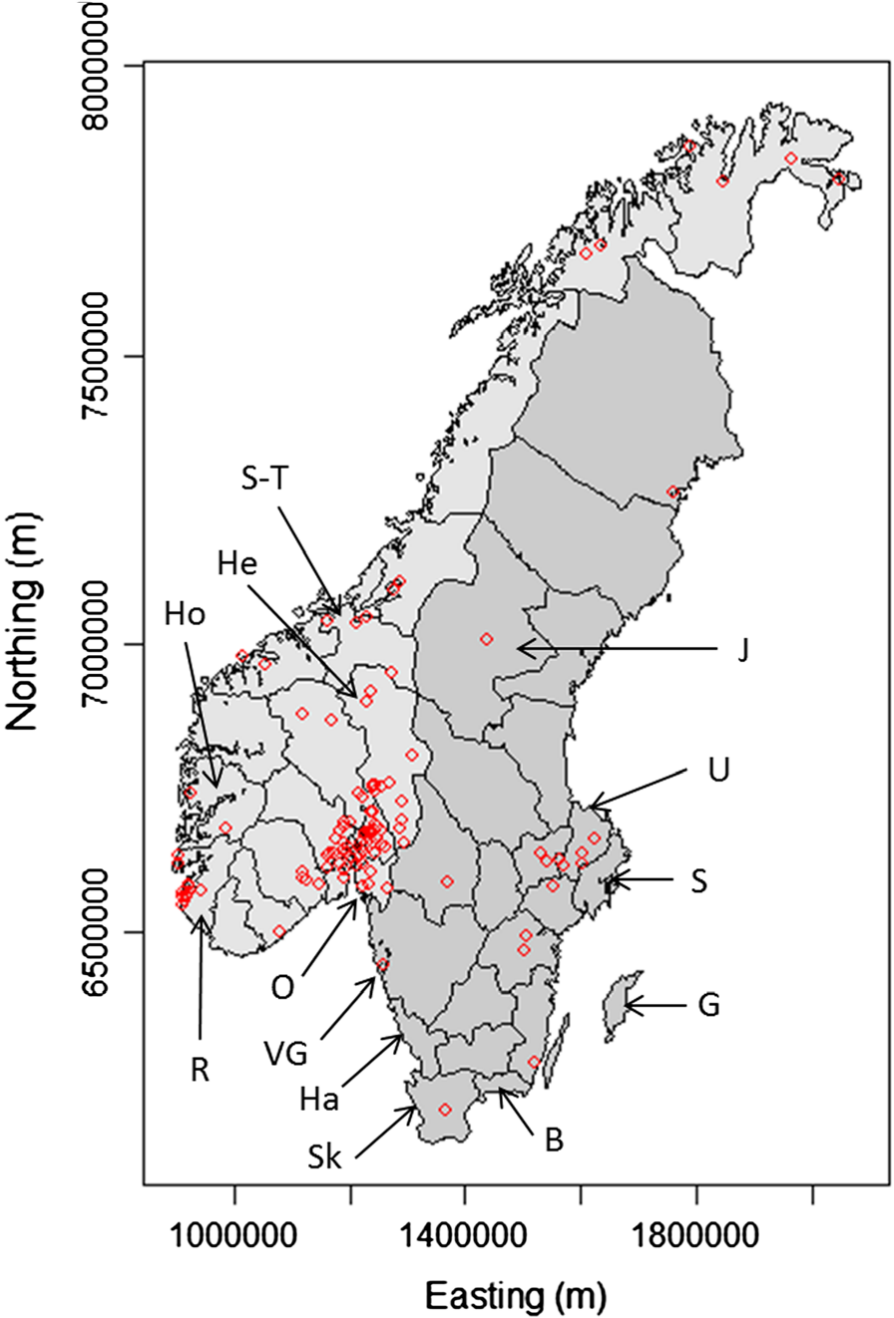
**Geographical locations of case farms with acquired equine polyneuropathy.** In a retrospective study of all farms with at least one reported case (n = 135) of acquired equine polyneuropathy in Norway (light shading) and Sweden (dark shading) in 1995–2012. The labels indicate the counties of Jämtland (J), Uppland (U), Stockholm (S), Gotland (G), Blekinge (B), Skåne (Sk), Halland (Ha), Västra Götaland (VG), Oslo (O), Rogaland (R), Hordaland (Ho), Hedemark (He), Sør-Trøndelag (S-T).

Geographically, 120 farms (88%) were located in Norway, and 16 farms (12%) in Sweden. The 118 farms where the exact numbers of case horses were available were located both in Norway (102 farms, 232 case horses) and Sweden (16 farms, 102 case horses). Case farms were more numerous in and around Oslo county (Norway), but also in Rogaland county in southwestern Norway as well as in Uppland and west of Stockholm in Sweden (Figure 2).

### Space-time description methods

#### Local detection of space-time interaction clusters

The sample size for the first scan statistic at case horse level was 118 farms. In the full study period 1995–2012 there were six significant (p < 0.001) clusters of high rates of cases, two of which involved only one case farm, and one cluster with low rate of cases (Table 1). No cluster included case farms from both Norway and Sweden. The earliest cluster started in October 1998 and the last cluster ended in January 2012. Of the 118 farms, 110 were in the spatial window of a cluster, and of these, 11 were part of a spatio-temporal cluster of high case rates. During the temporal length of these clusters there were between 11 and 25 observed cases per cluster, when 0.65–1.88 cases would have been expected to appear if there had not been any clustering. When the data instead were analysed for three 6-year study periods, with eleven, 26 and 80 case farms, respectively, in total 12 clusters were found with high rates and three with low rates. Nine of these clusters were from the period 2005–2012, and spatially five (out of 15) clusters included only one herd.

**Table 1:** **Clusters from a space-time scan statistic (space-time permutation model) of acquired equine polyneuropathy cases**

**Cluster centre: county (country)**	**Cluster radius (km)**	**Cluster period (month + year)**		**Number of case farms**		**Number of cases**		**p-value**
		**From**	**To**	**Spatial window**	**Spatio-temporal window**	**Observed**	**Expected**	
Hedemark (NO)	38	Oct 1998	Mar 1999	6	3	12	0.79	< 0.001
Uppland (SE)	39	Apr 1999	Apr 1999	4	2	12	0.65	< 0.001
Jämtland (SE)	0	Feb 2005	Feb 2005	1	1	25	1.88	< 0.001
Hordaland (NO)	343	Feb 2005	Aug 2006	90	0	0	27.75	< 0.001
Uppland (SE)	0	Mar 2005	Mar 2005	1	1	12	0.68	< 0.001
Skåne (SE)	272	Feb 2009	Apr 2009	3	2	11	1.20	< 0.001
Sør-Trøndelag (NO)	104	Apr 2011	Jan 2012	5	2	12	0.88	< 0.001

The scan was performed with a maximal temporal window of 50% of the study period and a maximum spatial window including 50% of the study population. The study included all cases (n = 334) from all farms (n = 118) of reported acquired equine polyneuropathy during 1995–2012 in Norway (NO) and Sweden (SE) for which the month of the first case and the number of diseased horses on the farm was known.

The sample size for the second scan statistic, at case farm level, was 135 farms. In the full study period there were no significant (p < 0.05) clusters of high rates of cases but one cluster with low rates of cases (p < 0.01). The temporal length of this cluster was March to May 2012. The spatial window included 27 Norwegian and 12 Swedish case farms. In this space-time cluster there were no observed cases when 9.8 cases were expected. Because of the lack of statistically significant clusters with high rates of cases the case farm level scan statistic was not repeated for each 6-year study period.

#### Global tests of space-time interaction clustering

The sample size for both these analyses was 135 case farms.

**Figure 3:**
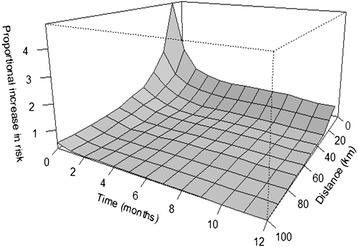
**Proportional increase in disease risk due to space-time clustering (D**_**0**_ **(s, t)) with the K-function.** The elevated surface illustrates the excess in risk for AEP within certain spatial and temporal distances. The retrospective study included all farms with at least one reported case of acquired equine polyneuropathy in Norway and Sweden during 1995–2012, for which the month of occurrence of the first case was known (n = 135).

The plot of the D_0_(s, t) from the space-time K-function analysis suggests an increase in disease risk, which was most prominent within distances of approximately 20 km spatially, and 2 months temporally (Figure 3). This suggests that space-time clustering was present in the data. Of the 999 simulated K-functions, only 1 had a higher value than observed, i.e. the p-value of the test statistic was < 0.002. When the analyses were repeated with the three 6-year study periods, the same pattern for D_0_(s, t) was found for the study periods 2001–2006 and 2007–2012; however, not for the earliest known cases in 1995–2000 (data not shown).

The Jacquez kNN test statistic was 3.0 (overall p-value 0.41). J_k_ and ΔJ_k_ were significant (p < 0.05) at the k = 3 level (Table 2) but there was no support for space-time clustering at lower levels of k. In other words, by definition, the kNN analysis did not support the presence of space-time clustering.

**Table 2:** **Results from the Jacquez k nearest neighbour (kNN) analysis for space-time clustering of case farms**

**k** ^**a**^	**J(k)** ^**b**^	**p(J(k))** ^**c**^	**ΔJ(k)** ^**d**^	**p(ΔJ(k))** ^**e**^
1	2	0.258	2	0.258
2	4	0.592	2	0.794
3	15	0.032	11	0.016
4	20	0.197	5	0.788
5	30	0.188	10	0.425
6	37	0.495	7	0.869
7	56	0.234	19	0.135
8	67	0.429	11	0.827
9	77	0.679	10	0.908
10	92	0.762	15	0.760

The overall combined (k 1–10) p-value was 0.128 using Sime’s correction (999 iterations were used to evaluate significance). The study included all farms with reported cases of acquired equine neuropathy in Norway and Sweden during 1995–2012, for which the month of occurrence of the first case was known (n = 135).

^a^Nearest-neighbour order.

^b^Number of space-time k nearest neighbours.

^c^Significance of J(k).

^d^Number of space-time k nearest neighbours when k was increased by 1.

^e^Significance of ΔJ(k).

In summary, the results from two of the three different methods of testing for space-time clustering of AEP cases supported, at least in part, the rejection of the null hypothesis of no space-time interaction.

## Discussion

### Temporal features

The variation in numbers of case farms across the different years included in the present study (Figure 1), as well as the high incidence of AEP in Norway in 2012, suggests that the factor(s) associated with the disease do not appear continuously with the same intensity. The clear seasonal pattern found suggests that horses are more likely to be exposed to the factor, or combination of factors, associated with AEP during a part of the year when they are not on pasture, assuming that the incubation period between exposure and development of clinical signs is not several months. An association between the number of horses of a particular breed and incidence of AEP is unlikely, which together with absence of breed predilection in other studies [3,4] again indicates that the causes of AEP are to be found in the horses’ environment rather than in a genetic predisposition. Moreover, case farms often had more than one affected horse, which, unless affected horses were related, is in favour of environmental exposure.

### Spatial features

One very interesting result was the large excess of case farms and cases in Norway compared with Sweden, despite the larger estimated Swedish horse population (Sweden, n = 362,700 horses in 2010; Norway, n = 125,000 horses in 2012) [22,23]. This geographical pattern of uneven case load was already noted in a previous study reporting AEP data from 2007–2009 [4], and remained in this study with all known cases of AEP from 1995 to 2012 included. Interestingly, all cases reported in 2010–2012 were from Norway. Management variables, meteorological variables or other factors with local or regional differences between horses, which could explain outbreaks of AEP remain to be hypothesised and studied, as further discussed below.

Further, the areas where many cases of AEP aggregated (Figure 2) correspond to some, but not all, areas where the human population is dense. This is not unexpected as three-quarters of the total horse population and two-thirds of all establishments with horses in Sweden are located either in city areas or in areas adjoining urban areas [23]. The three counties with largest subpopulations of horses in 2010 (approx. 50,000 horses each) are Stockholm, Västra Götaland and Skåne [23] (Figure 2) and 42% of all Swedish horses thus live there. However, the outbreaks of AEP in this study were not concentrated to these three regions, or even to the five most horse-dense counties (Stockholm, Skåne, Blekinge, Gotland, Halland, Figure 2), as might have been expected if risk factors had been evenly distributed across the whole horse population or if a dense horse population in itself would constitute a risk factor. Risk factors for AEP at the individual horse level have been discussed by Gröndahl et al. [4]. A farm with many horses could, obviously, have more cases than a farm with only a few horses. There were no data suggesting that farms with larger horse populations were located in certain areas or that such farms were affected during certain time periods, which, had this been the case, could possibly have explained the higher number of cases for some areas and during some periods.

### Spatio-temporal features

The distribution of case farms in time and space led us to form a hypothesis of space-time interaction clustering of AEP case farms (and cases). Space-time clustering was supported by the results from the spatial scan statistic at case level and at case farm level by the space-time K-function, although some clusters detected by the scan statistic included a larger area and longer time period than suggested by the distances with increased risk found with the K-function. Also, the proportion of case farms that were included in a space-time cluster was low (eleven out of 118). In this scan statistic, all case farms for which information on number of affected horses was missing were excluded, which is a limitation of the study. The second scan statistic, at case farm level, included these 18 farms, 17 of which were in Norway and affected in 2012; nine of these 17 farms were located in Rogaland County (Figure 2). However, no clusters of high rates of case farms were identified.

The first scan statistic evaluated if affected horses were clustered or not. The second scan statistic evaluated if affected farms were clustered or not adjusting for any within-farm clustering of case horses, i.e. that one horse would be more likely to be affected if there already was one affected horse on the farm. Although the number of affected horses per farm often was >1, the median was two and therefore the overall within-farm clustering could be regarded as low compared to many infectious diseases, Nevertheless, two of the clusters identified with the first scan statistic included only one farm each with many case horses. It would have been interesting to study exposure to potential risk factors in the farms identified by the first scan statistic to learn more about a potential aetiology of AEP and to compare to results from other studies of risk factors for AEP [4]. Further, an investigation of the case farms in the spatio-temporal cluster with fewer cases than expected, determining what possible exposures that were absent during this time period, would have been interesting.

Because of the lack of data on the background population, methods for only case-data had to be used to evaluate space-time interaction clustering. By using case-only methods, the interpretation of the results cannot be done in relation to the density of the horse population where clusters are detected, and the incidence will not be possible to estimate. Even though cases have been reported in less horse-dense areas, and despite that the true incidence is not known, we suggest that AEP should be considered a relatively rare disease. However, the long-term loss of performance and high mortality described [4] still merits the disease to be classified as a serious equine health problem in the areas affected and research for further knowledge on aetiology, prophylactic and therapeutic management is warranted.

The global tests of the overall spatio-temporal features of the case data produced different results. The positive space-time K-function uses the Euclidian distances between case locations, i.e. two farms are “close” if they are in the same geographical area. The negative kNN on the other hand, does not consider the distances between case locations, i.e. the neighbour case farm can be geographically located on the other side of the road or far away. This difference in what type of closeness the two methods evaluate could be one reason for the different results, and suggests that the exposure is geographically limited.

The local test for detection of space-time interaction clustering, i.e. the scan statistic, produced some clusters of a size that was, roughly, in agreement with the temporal distances of two months as well as the spatial distances of approximately 20 km seen in the test with the K-function (Figure 3), however three clusters had a considerable larger radius in kilometres (Table 1). Both the larger and smaller clusters detected with the scan statistic could possibly be attributed to factors such as natural geographical structures or barriers, shared network of feed suppliers, experienced similar climatic conditions, etcetera during the temporal length of the cluster. Using case-only methods also means the interpretation of the results cannot be done in relation to the density of the horse population where clusters are detected, and the incidence will not be not possible to estimate, although AEP should be considered a rare disease also when cases have been reported in less horse-dense areas. Nevertheless, AEP is relevant to study because of the high mortality and lack of knowledge of the aetiology.

Outbreaks of infectious diseases typically show space-time clustering when disease is spread from one herd or animal to its neighbours. But non-infectious disease may also appear in outbreaks if the aetiology is related to transient and local causes, e.g. environmental exposure. The findings suggests either a variable incubation time between exposure and development of clinical signs, or a multifactorial background, or else that the same exposure phenomenon may appear at various times of the year but with a strong predilection for certain periods of the year. Previous studies have discussed a forage-related aetiology for AEP [3,4], specifically use of wrapped forage; however, this hypothesis remains to be proved. The hypothesis is based on the fact that affected cases usually had been fed wrapped forage (haylage or silage), as reported in more than 95% of the case farms in the present study (data not shown). A shift from hay to wrapped forage occurred in many horse farms in Norway and Sweden in the 1990s, concurrent with the emergence of AEP in these countries. In the present study, there was no space-time interaction during the period 1995–2000, examined using the space-time K-function. The first case horse level scan statistic, on the other hand, did detect two clusters during this time period. However, the second scan statistic, at case farm level, did not. Many of the first affected case farms included in this study were early adaptors to the use of wrapped forage, and on some farms only horses fed a certain batch of wrapped forage become affected with AEP [4]. Today, wrapped forage is used partly or exclusively as roughage fed to horses during the winter by over 50% of horse keepers in Norway and Sweden [22,24], and in up to 90% of the bigger establishments in Sweden [24], but our data do not suggest a corresponding increase in the incidence of AEP, so factors other than simple exposure to wrapped forage have to be part of a “forage hypothesis”.

One environmental exposure possibly shared by case farms, and which may be coupled to forage quality, is the meteorological conditions during grass growth, at harvest, during storage time or during feed-out. Local meteorological conditions affecting pasture grass and thus triggering disease in susceptible animals have been discussed in relation to spatio-temporal clustering of cases of equine grass sickness [25]. Weather (including temperature, rainfall, air humidity) and sun irradiation at harvest largely influences the wilting rate of the cut crop and a slow wilting process may result in increased microbial growth or toxin production in the crop already before baling and wrapping. In one study, forage microbial load in wrapped forages on commercial horse farms in autumn and in the following spring did not show increased mould growth after winter storage [26]. Preliminary results from a study of forages in 13 farms affected with polyneuropathy suggest that weather conditions during forage harvest were unfavourable (wet and damp), and the forages often showed presence of soil contamination, grass roots and decaying plant material (personal communication, Gröndahl, G). Both findings are considered risk factors for a good hygienic quality of the end product. It would have been interesting to study risk factors in climatic conditions around the harvest date at the harvest location for the forage of each case farm in the present study, and also at the farm location at the time of the outbreak, but such background data were unavailable.

The three methods used to test for space-time clustering assume that the density of the background population is stable, or is changing at a rate that is consistent through space [13]. If this is not the case, this may cause bias where non-existent clusters of cases are detected. There was no information suggesting that the horse population in Norway or Sweden should have changed very differently in different regions during the study period. The population growth was assumed to be homogeneous in space and through time. The Swedish horse population was estimated to have increased by 10–20% from 2004 to 2010 in a study by the Swedish Board of Agriculture [23]. The spatial resolution was number of horses per county in Sweden. In Norway, the only survey conducted did not include the geographical distribution of the horse population [22].

To reduce bias caused by any heterogeneous changes in the background population, i.e. detection of non-existent clustering [27,28], the tests with positive results (space-time K-function and scan statistic at case horse level) were repeated with subsets of the data covering 6-year periods, or geographically including only southern Norway (K-function). By using three different methods, and in addition repeating some analyses for subsets of the data, the risk of type I error, i.e. incorrectly discarding H_0_ of no clustering, increased. However, the results that were positive were significant at a rather strict α-level. Further, the Jacquez kNN test included a correction for multiple testing.

A limitation of the study was the small sample size in relation to the size of the study area and length of the study period. This may have caused lack of power and increased risk of type II-error, and could explain why the scan statistic at case farm level and Jacquez kNN test result were negative. The statistical power of techniques to detect space-time clustering has been described as low to moderate [29], which in the present study could have led to false negative results.

With the passively reported case data, one possible reason for the higher caseload in Norway could be larger under-reporting in Sweden. During the last 6 years of the study period a research project, of which the current study is a part, has included a number of articles in magazines for veterinary practitioners and horse owners in both Norway and Sweden. Because of the typical clinical manifestation of AEP any veterinarian working in horse care should be able to diagnose the disease independently or at least after consulting a referral clinic. Nevertheless, there were likely cases of AEP that were never diagnosed, reported and included in this study and possibly this under-reporting was larger in some areas for unknown reasons. It has been shown that the space-time K-function was not invalidated by under-reporting if the under-reporting in time, e.g. during certain calendar months, was independent of the under-reporting in space, e.g. by a particular veterinary practice [25]. We reason it unlikely that the under-reporting in the present study should be linked to time or space, or that under-reporting is larger in Sweden than in Norway.

If geographic proximity to data collection centres in Oslo and Uppsala increased the inclination to consult on or report AEP cases, this might be a possible confounder for geographical clustering. Nevertheless, we think that larger outbreaks were recognised in the study regardless of location, because of the public awareness of the disease, the high degree of social networking in the equine community in Scandinavia, and the commotion in the community generally noted at outbreaks of this severe disease.

## Conclusions

The results suggest an aetiology of AEP where the exposure is not consistent in time, but varies during and between years. The results further suggest that the exposure also varies between regions. Outbreaks were more common in late winter and in spring, and more numerous in certain regions, which did not strictly correspond to the known horse population density. Spatio-temporal clustering of case farms was found with two out of three different methods. The inconclusive result may be explained by the relatively low number of cases and the low to moderate power of methods to detect space-time clustering when the background population is unknown. Further research is needed to study how management variables, meteorological variables or other factors with local or regional differences between horses may explain outbreaks of AEP.
